# Sialolithiasis: retrospective analysis of the effect of an escalating treatment algorithm on patient-perceived health-related quality of life

**DOI:** 10.1186/s13005-021-00259-1

**Published:** 2021-03-01

**Authors:** Julian Lommen, Lara Schorn, Benjamin Roth, Christian Naujoks, Jörg Handschel, Henrik Holtmann, Norbert R. Kübler, Christoph Sproll

**Affiliations:** 1 Department of Oral and Maxillofacial Surgery, Heinrich-Heine-University, Moorenstraße 5, 40225 Düsseldorf, Germany; 2 MKG Brühl, Uhlstraße 95-97, 50321 Brühl, Germany; 3 Clinic for Oral and Maxillofacial Surgery, Klinik am Kaiserteich, Reichsstraße 59, 40217 Düsseldorf, Germany; 4 Department of Oral and Maxillofacial Surgery, Evangelisches Krankenhaus Bethesda, Ludwig-Weber-Straße 15, 41061 Mönchengladbach, Germany

**Keywords:** Sialolithiasis, Submandibular gland, Parotid gland, Minimal-invasive, Algorithm

## Abstract

**Background:**

Gland preserving techniques in the treatment of sialolithiasis have continuously replaced radical surgery. The aim of this study was to evaluate a multimodal treatment algorithm in the therapy of sialolithiasis and assess improvement of HRQoL perceived by patients.

**Methods:**

Patients with sialolithiasis were treated by a multimodal treatment algorithm based on multiplicity of stones, stone size, affected gland, and stone position. The therapeutic spectrum ranged from conservative measures, extracorporeal shockwave lithotripsy, interventional sialendoscopy, combined endoscopic-surgical procedures to surgical gland removal as ultima ratio. Outcomes were evaluated by surgeons by means of the electronic patient record and by patients themselves using a standardized questionnaire.

**Results:**

87 patients treated for sialolithiasis were comprised in this study. The submandibular gland (SMG) was affected in 58.6% and the parotid gland (PG) in 41.4% of cases. Mean patient age was 41.67 years for SMG and 48.91 years for PG. In over 80% of cases sialolithiasis was associated with classic meal-related pain and swelling. Type and intensity of symptomatic sialolithiasis were not dependent on patient age or gender, nor could a relation between the affected gland and the occurrence of symptoms be demonstrated. Overall, 86.2% of cases were reported as cured using the multimodal step-by-step treatment algorithm. Resection of the affected gland could be dispensed in 98.9% of cases. According to patients pain could be reduced in 94.3% of cases.

**Conclusions:**

The analyzed treatment algorithm of increasing invasiveness is a favorable and effective tool to successfully treat sialolithiasis in > 86% of cases. For the first time, the present study shows that patient-perceived improvement of HRQoL due to ease of symptoms has an even higher success rate of > 94%.

**Supplementary Information:**

The online version contains supplementary material available at 10.1186/s13005-021-00259-1.

## Background

The most common cause of obstructive sialadenitis of the major salivary glands is sialolithiasis [1]. This disease is characterized by formation of calcified stones (sialoliths) within the gland’s ductal system that hinder saliva outflow into the oral cavity. Etiologically, changes in ion composition, quantity and flow rate as well as pH changes of the saliva, nicotine abuse and dehydration are currently being discussed [2, 3]. The incidence of sialolithiasis within the general population is estimated to be between 28 and 59 cases per million and year [2]. The mean age for onset of symptoms due to sialolithiasis is approximately 45 years [4]. Of all major salivary glands the submandibular gland (SMG) is specifically prone to sialolith formation due to its seromucous saliva content as well as a long and curved duct (Wharton’s duct) both of which facilitate calcification [5]. The parotid gland (PG) is the second most affected gland followed by the sublingual gland (SLG) which rarely is affected [6]. The average time between the occurrence of symptoms like recurrent colicky pain and swelling of the affected gland and final diagnosis is 2.4 years [7]. Without removal of the obstructive sialolith complications like abscesses, fistulas and phlegmonous inflammations have been reported [8]. Cervical sonography is the gold-standard modality for diagnosis of sialolithiasis due to its ubiquitous availability, low cost and non-invasiveness [9]. The specificity and sensitivity of sonography in sialolith detection is reported to be 94 and 86%, respectively [10]. In the 1990s the procedure of diagnostic sialendoscopy was introduced as a method to directly visualize sialoliths by insertion of a semi-rigid endoscope with a diameter of no more than 1.7 mm into the gland’s excretory duct [11]. Additionally, sialography, computer tomography (CT), cone-beam computer tomography (CBCT) as well as magnetic resonance sialography (MRS) can be conducted to diagnose sialolithiasis [12]. The line of therapy follows an escalating treatment algorithm depending on the type of affected gland as well as the position and number of sialoliths [13, 14]. Due to the deep position within the ductal system sialolith formation in the hilus and the parenchyma is most difficult to treat [4]. Whereas sialadenectomy was often conducted in these cases the innovative guidance of the developed treatment algorithm nowadays allows gland preservation in > 90% of cases leading to lower postoperative complication rates such as wound infection and injuries to the lingual and facial nerve [4, 15]. According to the treatment algorithm initial prescription of antibiotics in combination with analgesics, sialogogues and massage of the affected gland should be attempted to enable spontaneous sialolith discharge [12, 13]. Sialoliths of the SMG and PG with a diameter of no more than 3 mm can usually be removed with sialendoscopy with success rates of > 90% [10, 16]. Larger sialoliths within the distal third of Wharton’s duct can be removed by papillotomy, whereas sialoliths within the middle third are commonly extracted by sialendoscopy [17]. Sialoliths within the parenchyma of SMG and PG that cannot be removed by interventional sialendoscopy (ISE) or extracorporeal shockwave lithotripsy (ESWT) alone may be removed by an intraoral (SMG) or extraoral (PG) endoscopy-assisted sialolithotomy (IEAS) with success rates of > 90% [18, 19]. As a last resort in rare cases with inaccessible symptomatic sialoliths sialadenectomy has to be performed.

The aim of the present study was to retrospectively analyze and evaluate the therapeutic success rates of an escalating treatment algorithm in sialolithiasis. The novelty in this study is the special emphasis on the patient perceived physical and psychological strain throughout therapy.

## Methods

### Ethical approval

Approval of the Ethics Committee of Düsseldorf University Hospital was granted prior to conducting this retrospective study and given the study number 2019–632.

### Patient collective

Overall, 110 patients with either radio- or sonographically diagnosed sialolithiasis of the SMG or PG were treated at the Department of Oral and Maxillofacial Surgery at Düsseldorf University Hospital between January 2013 and July 2018. After adaptation to the inclusion criteria of age ≥ 18 years and a minimal duration of ≥6 months between intervention and time of follow-up 87 patients could be included in the study.

### Treatment algorithm

**Fig. 1:**
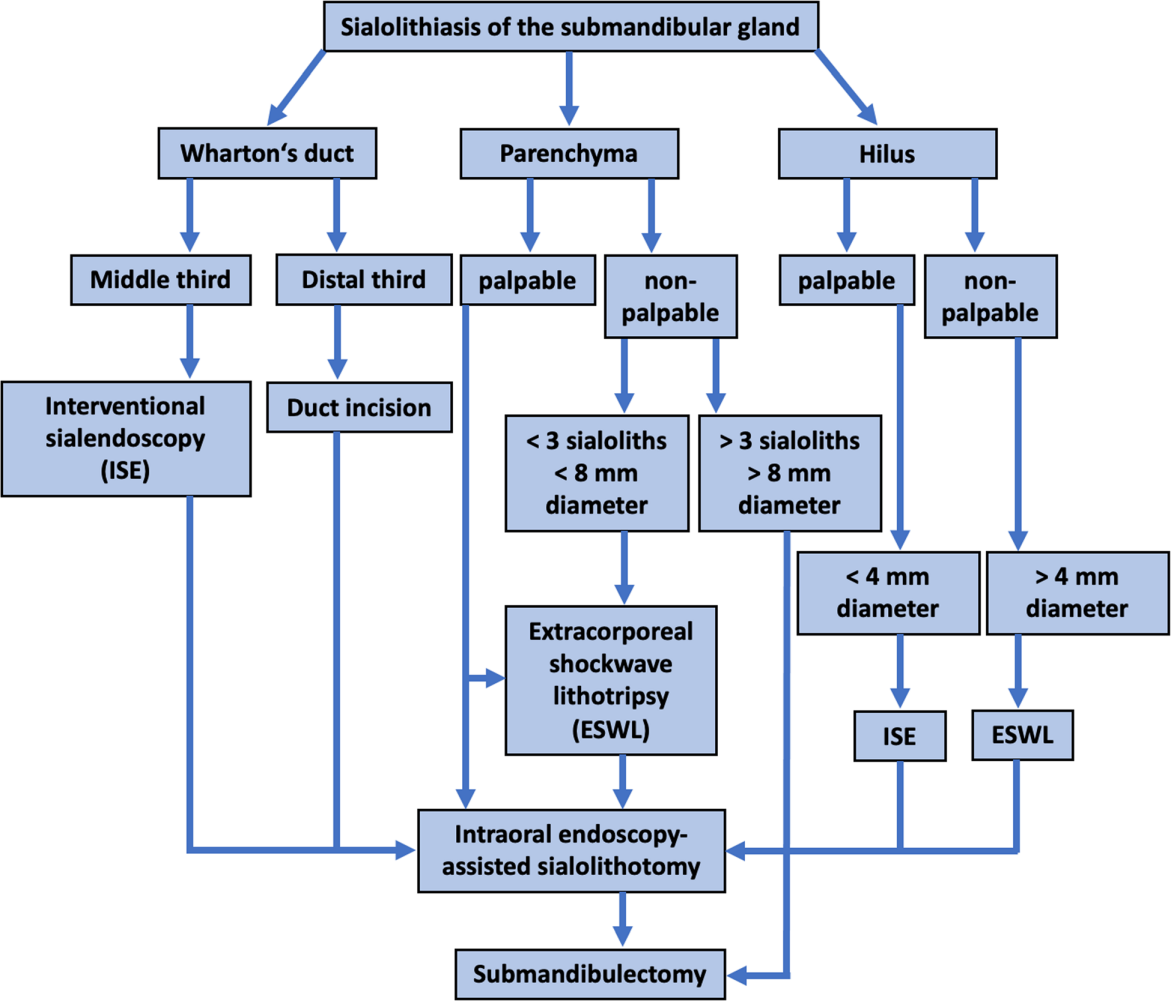
Minimally-invasive treatment algorithm for therapy of sialolithiasis of the submandibular gland (SMG) (modified according to Koch et al., 2009) [20]

Based on the multicenter study by Iro et al. (2009) with 4691 patients which describes a reduction in conducted sialadenectomies due to sialolithiasis to merely 2.9% by means of extracorporeal shock-wave lithotripsy, sialendoscopy and gland preserving submandibular and parotid surgery as well as a study by Koch et al. (2009) we established a modified escalating treatment algorithm [4, 20]. These modifications were based on the authors’ surgical experience and detailed research of contemporary literature. Treatment modalities for the SMG were adjusted according to the anatomical stone position in (1) Wharton’s duct, (2) hilus and (3) parenchyma (Fig. 1).

Stones located within the distal third of Wharton’s duct were retrieved by duct incision, whereas stones in the middle third of Wharton’s duct were removed by ISE. In cases were stones could not be removed in either of the aforementioned procedures IEAS or submandibulectomy were conducted. Palpable stones within the hilus region measuring < 4 mm in diameter were treated by ISE, whereas non-palpable stones measuring > 4 mm were treated by ESWT. Subsequently, IEAS or submandibulectomy was the treatment of choice when ISE and ESWT failed. Palpable stones within the gland’s parenchyma were treated by IEAS or submandibulectomy as ultima ratio. Submandibulectomy as performed for > 3 stones or a single stone with a diameter of > 8 mm within the parenchyma (Fig. 1).

**Fig. 2:**
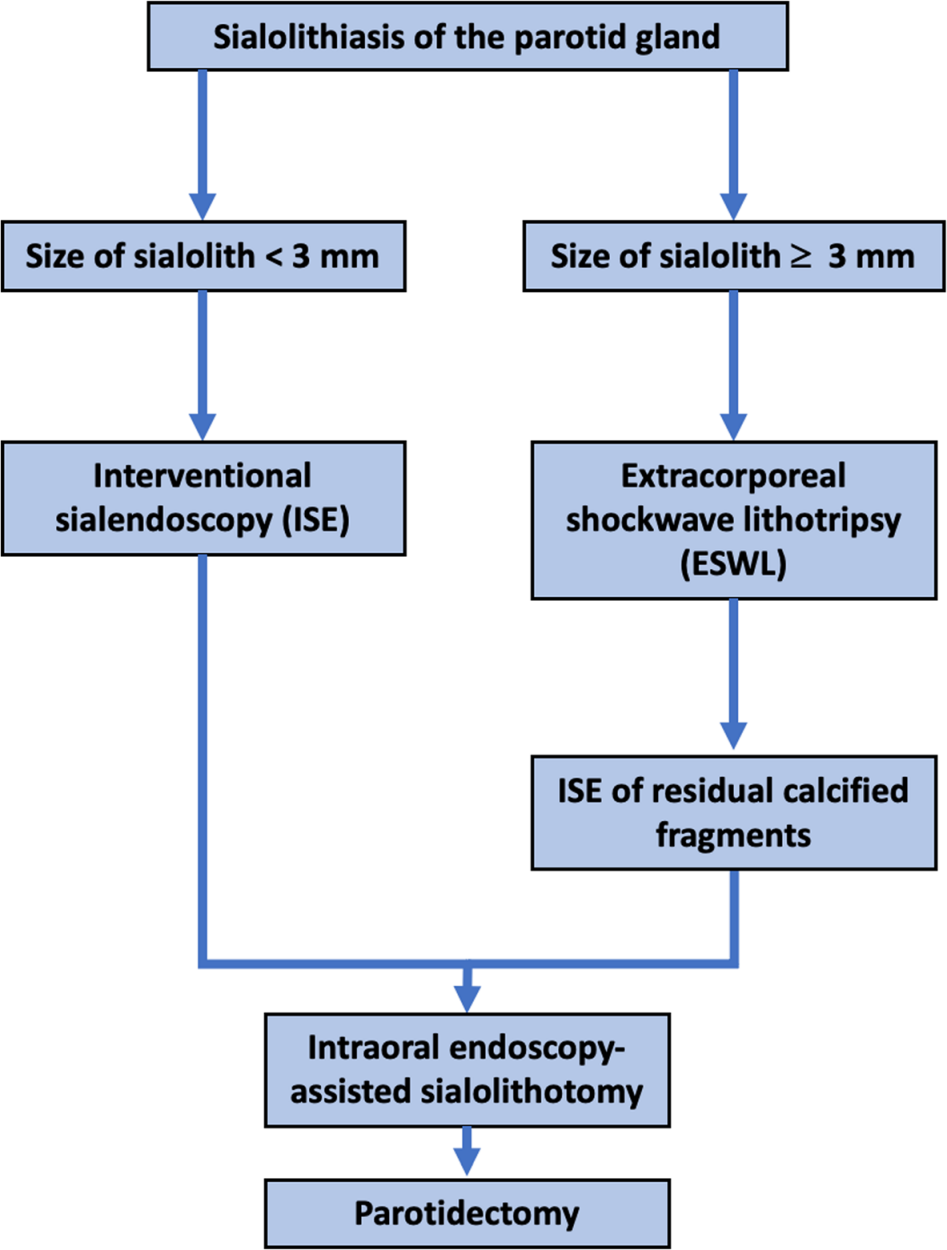
Minimally-invasive treatment algorithm for therapy of sialolithiasis of the parotid gland (PG) (modified according to Koch et al., 2009) [20]

Stones of the PG < 3 mm in diameter were treated by ISE and in case of failure followed by IEAS or lateral parotidectomy (Fig. 2). Larger stones with > 3 mm in diameter were treated by ESWT alone or in combination with ISE (Fig. 2). Again, IEAS or lateral parotidectomy had to be conducted as ultima ratio.

The modifications to the suggested treatment algorithm by Koch et al. (2009) applied in this study are based on our clinic’s standard operating procedures (SOP) for sialolith therapy (Figs. 1 and 2) [20].

All interventions were conducted by the same trained oral maxillofacial surgeon and the analysis was also conducted by another but also always the same assessor to reduce bias.

### Data acquisition

Retrospectively, patient data such as age, gender, medical history, affected gland, type of symptoms as well as the type of intervention were retrieved from the patient’s medical file. Additionally, a standardized questionnaire was filled out by patients before (Tab. 1), directly after (Tab. 2) and ≥ 6 months after (Tab. 3) the intervention.

### Statistics

Statistical analysis was conducted with “Statistical Package for the Social Sciences” (SPSS, IBM, version 24) software for Mac as well as Microsoft® Excel Version 2016 (Microsoft® Excel, California, USA) for analyzes of patient perceived symptoms on a numeric rating scale. Data are described as means and standard deviation (SD). A *p*-value < 0.05 was considered statistically significant.

## Results

### Population cohort

Mean patient age was 45.24 years with the youngest patient being an 18-year-old woman and the oldest a 92-year-old male. The mean patient age at time of initial presentation with symptomatic sialolithiasis was 41.67 years (SD = 17.079) for SMG and 48.91 years (SD = 16.466) for PG. These differences were statistically significant (*p* < 0.03). Of all 87 patients 44 (50.6%) were male and 43 (49.4%) female. Women had a mean age of 44.86 years (SD = 17.085) and men presented with a mean age of 45.48 years (SD = 17.327). These differences were statistically not significant (*p* > 0.05).

### Affected glands and symptoms

The SMG was affected by sialolithiasis in 51 (58.6%) cases, whereas the PG was affected in merely 36 (41.4%) cases. The left SMG was affected in 31 (54.4%) and the right in 20 (45.6%) cases. For the PG frequencies of sialolith occurrence were 21 (58.3%) for the right and 15 (41.7%) for the left side. These differences were statistically not significant (p > 0.05). Symptoms were grouped in four categories; (1) swelling, (2) pain, (3) swelling and pain and (4) no symptoms. Three (5.9%) patients with SMG sialolithiasis as wells as three (8.3%) patients with PG sialolithiasis were found in category 1. Seven (13.7%) patients with SMG sialolithiasis and four (11.1%) patients with PG sialolithiasis were found in category 2. Swelling and pain (category 3) was most frequently observed with 42 (82.4%) in patients with SMG sialolithiasis and 29 (80.6%) patients in PG sialolithiasis. We did not find any asymptomatic patients (category 4) as treatment was only conducted for symptomatic sialolithiasis at our clinic. Type and intensity of symptomatic sialolithiasis were not dependent on patient age or gender. No significant differences could be shown (*p* > 0.05). Furthermore, no significant differences between the affected gland and the occurrence of symptoms could be shown (p > 0.05).

### Therapeutic interventions in SMG sialolithiasis

**Fig. 3:**
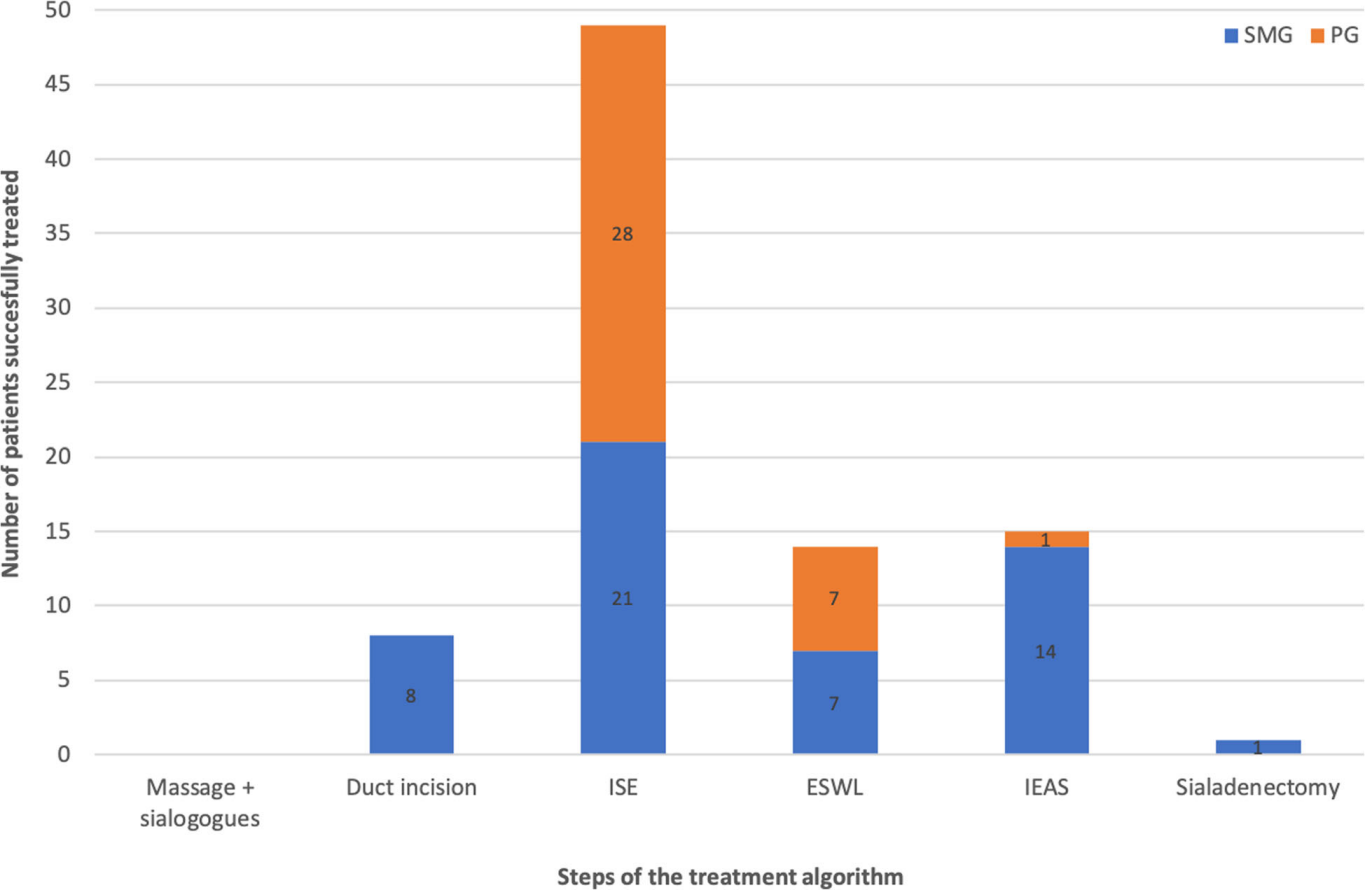
Number of successfully treated patients per step of the escalating treatment algorithm for submandibular gland (SMG) [blue] and parotid gland (PG) [orange]. From left to right: (1) conservative treatment by massage and sialogogues, (2) incision of Wharton’s duct, (3) interventional sialendoscopy (ISE), (4) extracorporeal shockwave lithotripsy (ESWT), (5) intraoral endoscopy-assisted sialolithotomy (IEAS) and (6) sialadenectomy

Gland massaging and use of sialogogues as the first step of the treatment algorithm was conducted by all patients upon instruction. In no case of this study was this modality sufficient to remove the stone from the SMG. In eight (15.7%) cases successful stone removal could be achieved by duct incision. In 21 (41.2%) cases ISE was conducted to successfully remove the stone. In 14 (27.5%) cases IEAS was applied to retrieve the sialoliths. In seven (13.7%) cases ESWT was used to successfully treat SMG sialolithiasis. One (2.0%) patient had to be treated by submandibulectomy for all other treatment options failed in retrieving the stone. Hence, only one patient had to undergo all steps of the treatment algorithm resulting in submandibulectomy (Fig. 3). Indication for use of the aforementioned treatment modality was decided on according to the modified treatment algorithm for sialolithiasis of the SMG (Fig. 1). 40 (78.4%) patients only had to undergo one intervention, nine (17.6%) patients had two and two (3.9%) patients underwent three interventional approaches to eventually remove the stone. All patients who underwent two or more interventional approaches reported unease with the long duration of therapy until finding the suitable treatment modality.

### Therapeutic interventions in PG sialolithiasis

Gland massaging and use of sialogogues as the first step of the treatment algorithm was also conducted by all patients upon instruction. In no case of this study was this modality sufficient to remove the stone from the PG. In no cases successful stone removal could be achieved by duct incision. In 28 (77.8%) cases ISE was conducted to successfully remove the stone. In one (2.8%) cases IEAS was applied to retrieve the sialolith by means of an extraoral incision. In seven (19.4%) cases ESWT was used to successfully treat PG sialolithiasis. No patient had to be treated by lateral parotidectomy. Hence, no patient had to undergo the entire treatment algorithm to remove the sialolith (Fig. 3). Indication for use of the aforementioned treatment modality was decided on according to the modified treatment algorithm for sialolithiasis of the PG (Fig. 2). 29 (80.6%) patients only had to undergo one intervention and seven (19.4%) patients had two interventional approaches to eventually remove the stone. All patients who underwent two interventional approaches reported unease with the long duration of therapy until finding the suitable treatment modality.

### Analysis of the therapy success in SMG sialolithiasis

Evaluation of therapy success was divided in four groups; (1) cured (stone-free and symptom-free), (2) partial success (residual calcified fragments and symptom-free), (3) partial failure (stone-free and persisting symptoms) and (4) failure (residual calcified fragments and persisting symptoms). By this definition 45 (88.2%) patients could be cured (group 1). Three (5.9%) patients were categorized in group 2 and three (5.9%) patients were found in group 3. In this study no patient was found in group 4.

### Analysis of the therapy success in PG sialolithiasis

Evaluation of therapy success in PG sialolithiasis was also divided in four groups; (1) cured (stone-free and symptom-free), (2) partial success (residual calcified fragments and symptom-free), (3) partial failure (stone-free and persisting symptoms) and (4) failure (residual calcified fragments and persisting symptoms). By this definition 30 (83.3%) patients could be cured (group 1). Four (11.1%) patients were categorized in group 2 and two (5.6%) patients were found in group 3. In this study no patient was found in group 4.

### Development of symptoms in the course of the therapy

The degree of symptoms could be eased in 82 (94.3%) cases. 5 (5.7%) patients did not report ease of symptoms within the 6 month follow-up period. Overall, the treatment algorithm for therapy of sialolithiasis significantly (*p* < 0.005) eased symptoms from a mean pain intensity of 60 (SD = 8.65) (NAS) before the intervention to a mean pain intensity of < 10 (SD = 4.32) (NAS) after the intervention.

### Assessment of tolerability of the different interventions

In terms of patient unease and pain during diagnostic and interventional sialendoscopy, ESWT, papillotomy, intraoral endoscopy-assisted sialolithotomy and submandibulectomy no significant differences could be detected.

### Patient satisfaction

Overall, patient satisfaction with the escalating treatment algorithm of sialolithiasis was rated with 92 out of 100 possible points. 94.3% of patients would choose the same therapy approach again.

## Discussion

In the last 15 years a paradigm shift in sialolithiasis treatment became obvious which favors gland preservation in most cases to avoid facial nerve lesions [21, 22]. Depending on the type of affected gland therapy follows a minimally-invasive treatment algorithm [20]. Studies reported improved health-related quality of life (HRQoL) of sialendoscopy despite higher costs [23]. In the present study we found a male:female ratio of 1:1 which is in line with the findings by Zenk et al. (1999) [7]. At initial symptomatic manifestation of sialolithiasis we found women to have a mean age of 44.86 years and men of 45.48 years. These data are comparable with other studies [4, 7]. In this study the SMG was affected in 58.6% of cases. In a study by Andretta et al. (2005) the SMG was affected in 92% of cases which significantly differs from our finding [24]. One reason for such discrepancy could be the smaller patient collective of our study with only 87 patients. On average patients with sialolithiasis of the SMG were 41.67 years old, whereas patients with obstructed PG were 48.91 years old. These findings are in accordance with data shown by other studies [7]. Pain and swelling were the most common symptoms of sialolithiasis in the present and comparable studies [25]. In the present study no patient presented with asymptomatic sialolithiasis. Other studies report significantly higher numbers [7]. This can be explained as only symptomatic patients were comprised in this study. Asymptomatic sialolithiasis usually does not require treatment. 86.2% of patients could be cured in the present study. Other studies report success rates of 75.5, 80.5 and 88.9% [1, 4, 26]. To patients, loss of pain and swelling of the affected gland was most relevant, whereas asymptomatic residual sialoliths posed only minor concerns. This partially explains the difference between patient perception of the term cured (94.3% of cases) and the surgeons perception (86.2%). These results can be explained by the good overall patient-perceived tolerability of sialendoscopy, especially, also shown by other studies [27]. In the present study parotidectomy could be omitted in 100% of patients and submandibulectomy was only necessary in one patient. These results may be due to the small number of patients included in the study but also point to the possibility that the treatment algorithm works better for parotid stones. Recurrence of sialolithiasis was more common for the PG than the SMG. Although the present study did not comprise patients who suffered from chronic sclerosing salivary gland inflammation caused by sialolithiasis we suggest the same treatment algorithm for this disease as it was shown by Marchal et al. (2001) that histological restoration of the gland’s sclerotic parenchyma to a physiological appearance can be achieved in some cases [28]. In cases of persisting symptomatic sclerotic sialadenitis gland resection may be a final option. On average, success rates of the step-by-step approach in sialolithiasis are reported with 84.23% in the current literature which is comparable to our finding with 86.2% [18, 19, 29, 30]. Sialolith removal from the PG was more tolerable to patients than from the SMG. The different therapeutic modalities like papillotomy, interventional sialendoscopy, ESWT and intraoral sialolithotomy were regarded as more tolerable than the symptoms of sialolithiasis themselves by patients. Contemporary studies describe holmium laser-assisted lithotripsy as an additional promising therapy for sialolithiasis [31]. Best outcomes using this technique were reported for midsize sialoliths with a diameter between 4 and 8 mm [32]. Generally, increased risks of damage to the excretory duct were not found to be higher using intraductal laser therapy compared to other treatment modalities [33]. It would be interesting to include laser lithotripsy to the presented treatment algorithm for future trials. However, currently a holmium laser is not available at our clinic. The treatment algorithm could significantly ease symptoms. This could also be shown by other studies [23, 34–37]. 94.3% of patients would want to receive the same therapy again in case of recurrent sialolithiasis which supports improved HRQoL.

## Conclusion

Treatment of sialolithiasis using a minimally-invasive treatment algorithm is a promising, well-established method to omit gland resection. The present study shows that patient-perceived improvement of HRQoL due to ease of symptoms has even higher success rates than stone removal alone.

## Supplementary Information

**Additional file 1: Table S1** Questionnaire to patients before the intervention.

**Additional file 2: Table S2** Questionnaire to patients directly after the intervention.

**Additional file 3: Table S3** Questionnaire to patients ≥6 months after the intervention.

## Data Availability

The datasets used and/or analysed during the current study are available from the corresponding author on reasonable request.

## Abbreviations

SMG: Submandibular gland
PG: Parotid gland
SLG: Sublingual gland
CBCT: Cone-beam computed-tomography
ISE: Interventional sialendoscopy
IEAS: Intraoral endoscopy-assisted sialolithotomy
ESWL: Extracorporeal shock wave lithotripsy
HRQoL: Health-related quality of life
