# Supplementary material for: Sialolithiasis: retrospective analysis of the effect of an escalating treatment algorithm on patient-perceived health-related quality of life
Source: Head Face Med. 2021 Mar 1;17:8. doi: 10.1186/s13005-021-00259-1 (PMC7919083; doi:10.1186/s13005-021-00259-1)
Supplement: Supplementary file 1 — Additional file 1: Table S1 Questionnaire to patients before the intervention. [file 13005_2021_259_MOESM1_ESM.docx]

**Table 1: Questionnaire to patients before the intervention**

| **Question to the patient** | |
| --- | --- |
| 1 | How often did symptoms occur in the past? (please only choose one option)   1. at rest  2. whenever ingesting food  3. daily  4. several times a week  5. weekly  6. monthly  7. semi-annual  8. never  |
|  |  |
| 2 | Did you have pain within the affected glandular region?  yes no |
| 3 | If yes, please grade pain intensity on a numeric analog scale from 1 (=no pain) to 100 (=maximum pain):  1 10 20 30 40 50 60 70 80 90 100 |
| 4 | Did you notice a gland swelling while ingesting food?  yes no |
| 4 | Do you suffer from a dry mouth (xerostomia)?  yes no |
| 5 | Do you have recurrent infections of the salivary glands?  yes no |
| 6 | Do you have other symptoms which are not stated above? Please provide in free text below: |
