# Supplementary material for: Sialolithiasis: retrospective analysis of the effect of an escalating treatment algorithm on patient-perceived health-related quality of life
Source: Head Face Med. 2021 Mar 1;17:8. doi: 10.1186/s13005-021-00259-1 (PMC7919083; doi:10.1186/s13005-021-00259-1)
Supplement: Supplementary file 2 — Additional file 2: Table S2 Questionnaire to patients directly after the intervention. [file 13005_2021_259_MOESM2_ESM.docx]

**Table 2: Questionnaire to patients directly after the intervention**

| **Question to the patient** | |
| --- | --- |
| 1 | How have you perceived the first intervention on a numeric rating scale from 1 (=very good) to 100 (=very bad)? Please provide the applicable type of intervention and numeric rating in free text below:  Type of intervention: Rating (1-100): |
|  |  |
| 2 | How have you perceived a possible second intervention on a numeric rating scale from 1 (=very good) to 100 (=very bad)? Please provide the applicable type of intervention and numeric rating in free text below:  Type of intervention: Rating (1-100): |
| 3 | How have you perceived a possible third intervention on a numeric rating scale from 1 (=very good) to 100 (=very bad)? Please provide the applicable type of intervention and numeric rating in free text below:  Type of intervention: Rating (1-100): |
